# Management of TAVI Underexpansion with Self-Expanding Valves: A Practical Approach

**DOI:** 10.3390/jcdd12060215

**Published:** 2025-06-07

**Authors:** Rene Hameau, Marco B. Ancona, Vittorio Romano, Luca Ferri, Barbara Bellini, Filippo Russo, Ciro Vella, Christos Papageorgiu, Francesca Napoli, Marco Licciardi, Gianluca Ricchetti, Andrea Tripoli, Rocco Sabarese, Lorenzo Zaccaria, Matteo Montorfano

**Affiliations:** 1 Interventional Cardiology Unit, IRCCS San Raffaele Scientific Institute, 20132 Milan, Italy; dr.renehameau@gmail.com (R.H.); romano.vittorio@hsr.it (V.R.); ferri.luca@hsr.it (L.F.); bellini.barbara@hsr.it (B.B.); russo.filippo@hsr.it (F.R.); vella.ciro@hsr.it (C.V.); chrispapageorgio@gmail.com (C.P.); napoli.francesca@hsr.it (F.N.); licciardi.marco@hsr.it (M.L.); richetti.gianluca@hsr.it (G.R.); tripoli.andrea@hsr.it (A.T.); sabarese.rocco@hsr.it (R.S.); zaccaria.lorenzo@hsr.it (L.Z.); montorfano.matteo@hsr.it (M.M.); 2 Unidad de Medicina Cardiovascular Integrada, Hospital Las Higueras, Talcahuano 426000, Chile; 3 Faculty of Medicine, Vita Salute San Raffaele University, 20132 Milan, Italy

**Keywords:** TAVI, infolding, underexpansion

## Abstract

Underexpansion of a self-expanding transcatheter aortic valve (TAVI) is a critical issue that can negatively impact long-term outcomes, including paravalvular leak, valve thrombosis, and increased mortality. This paper provides a comprehensive review of the pathophysiology and consequences of such complications, including three primary mechanisms: (1) infolding, (2) incorrect site of crossing and (3) true underexpansion. It also discusses strategies to address these challenges, including pre-procedural planning and procedural techniques to ensure proper valve deployment and expansion. Mitigating these issues is essential to improving both immediate and long-term outcomes in TAVI patients.

## 1. Introduction

Transcatheter aortic valve implantation (TAVI) for the management of severe aortic stenosis offers an effective and less invasive alternative to traditional surgical valve replacement. However, despite the substantial advances in technique and prosthesis design, complications still remain a significant challenge [1].

Self-expanding valves (SEVs) represent an evolving field with different devices available and showing unique features. This includes models with both supra-annular leaflets like the Evolut (Medtronic, Minneapolis, MN, USA) or the Acurate Neo (Boston Scientific, Marlborough, MA, USA) or intra-annular devices like the Navitor (Abbott, North Chicago, IL, USA). Most SEVs can be either repositioned and/or retrieved, offering a larger effective orifice area, reduced transvalvular gradient and decreased incidence of severe prosthesis–patient mismatch compared to intra-annular valves. Some limitations in maneuverability, especially in horizontal aortas, or coronary re-access with some devices along with a lower radial force are important factors to consider while choosing the correct transcatheter heart valve (THV) for each patient.

TAVI underexpansion refers to the failure of the transcatheter valve to fully expand during deployment, potentially leading to suboptimal hemodynamic results, clinical complications and impaired long-term durability. In this review, we explore the mechanisms underlying THV underexpansion, discuss its clinical implications and examine current strategies for prevention, detection and management. Understanding the underlying pathophysiology is essential for optimizing procedural outcomes and improving long-term prognosis.

## 2. Definition and Clinical Implications

While there is no universally accepted definition in the medical literature, underexpansion in TAVI procedures generally refers to the incomplete expansion of the THV stent frame, which fails to reach its intended nominal diameter during deployment.

With balloon-expandable THVs, circularity is relatively preserved, often achieving over 90% of expansion [2], but due to their lower radial force, SEVs frequently develop deformation and eccentric underexpansion at the inflow level [3]. This has been shown to impact valve functionality and durability by increasing leaflet stress and strain. It has been suggested that 5–10% of underexpansion can be clinically tolerable [4], but when this deformation extends into the functional portion, it can result in impaired valve motion or increased risk of leaflet thrombosis. Fuchs et al. [5] showed that moderate-to-severe regional THV underexpansion was associated with a significantly higher incidence of leaflet thickening than in cases of full regional THV expansion (24% vs. 3%, *p* < 0.01). They hypothesize that incomplete stent frame and leaflet expansion may result in the “wrinkling” of the leaflet as found in in vitro tests, making it more prone to the development of a thin thrombus layer on its surface [6].

Underexpansion also is associated with the presence of residual paravalvular leak (PVL), which has been consistently related to adverse events [7].The five-year follow up from the PARTNER-1 trial showed that moderate-to-severe aortic regurgitation after TAVI was associated with lower survival (HR 1.46, CI: 1.03–2.07, *p* = 0.04) [8]. In a prospective registry by Okuno et al., even mild paravalvular regurgitation after TAVI had a higher mortality risk at five years than those with none/mild leak (54.6% vs. 43.8%; HR adjusted 1.26, 95% CI: 1.06–1.50) [7].

Special consideration must be given when using SEVs with lower radial force, as pre-dilatation is highly encouraged by the manufacturer and post-dilatation is often needed to optimize the hemodynamic results. Recently, the ACURATE IDE trial [9] missed its non-inferiority primary endpoint when comparing the ACURATE neo2 (Boston Scientific) vs. the Evolut (Medtronic) and Sapien 3 (Edwards Lifesciences) platforms. At 1 year, the combined rate of all-cause mortality, stroke, or rehospitalization was 16.16% in patients treated with ACURATE neo2 and 9.53% among those treated with the other transcatheter valves. A post hoc analysis showed that underexpansion of the Acurate Neo 2 platform was present in nearly 20% of those devices, and when comparing underexpanded versus fully expanded valves, the first group showed numerically higher 1-year rates of hospitalization (18.8% vs. 12.4%; *p* = 0.050), death (7.4% vs. 3.7%; *p* = 0.054) and stroke (11.0% vs. 3.5%; *p* < 0.001). Interestingly, the fully expanded Acurate Neo 2 showed similar rates of adverse events when paired against the other two platforms.

## 3. Mechanisms of TAVI Underexpansion and Management

The mechanisms underlying underexpansion are complex and multifactorial, involving both patient-specific anatomical features and technical aspects of the procedure.

Understanding these factors is crucial for both prevention and management. In this section, we will review the key elements contributing to this complication and discuss strategies for optimizing the procedure, including pre- and post-deployment measures, aimed at mitigating its clinical impact.

### 3.1. Valve Infolding

Although it is now a rare complication affecting nearly 0,7% of implants with a second-generation Evolut platform [10,11], infolding can lead to severe consequences such as an increased risk of PVL, hemodynamic collapse, peri-procedural stroke and death [10,12]. This term describes the inward folding of the stent frame of an SEV along the vertical axis starting from the valve inflow.

The first preventive measure to avoid infolding is to inspect the valve before implantation to ensure proper loading [13].

**Figure 1:**
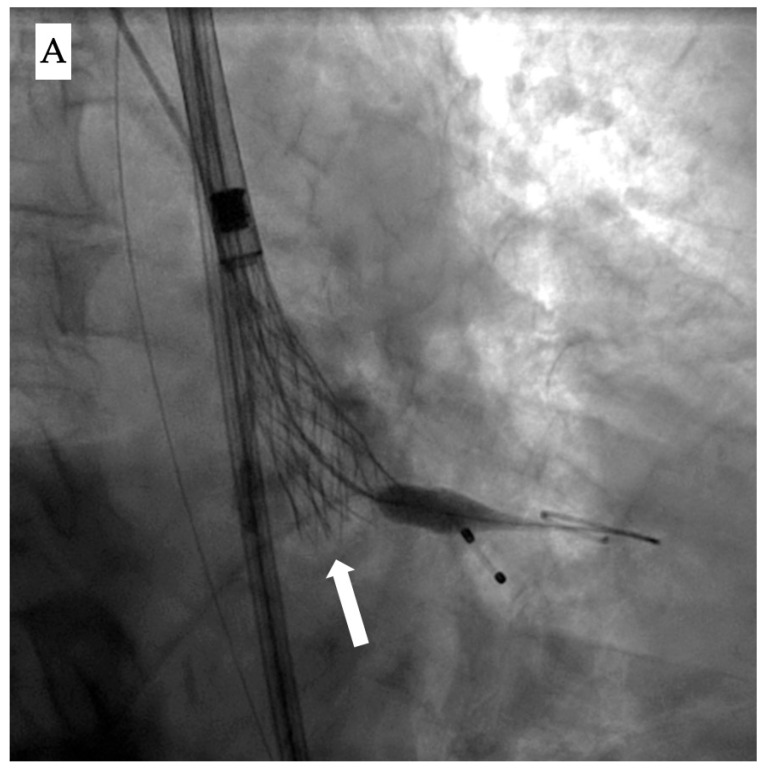

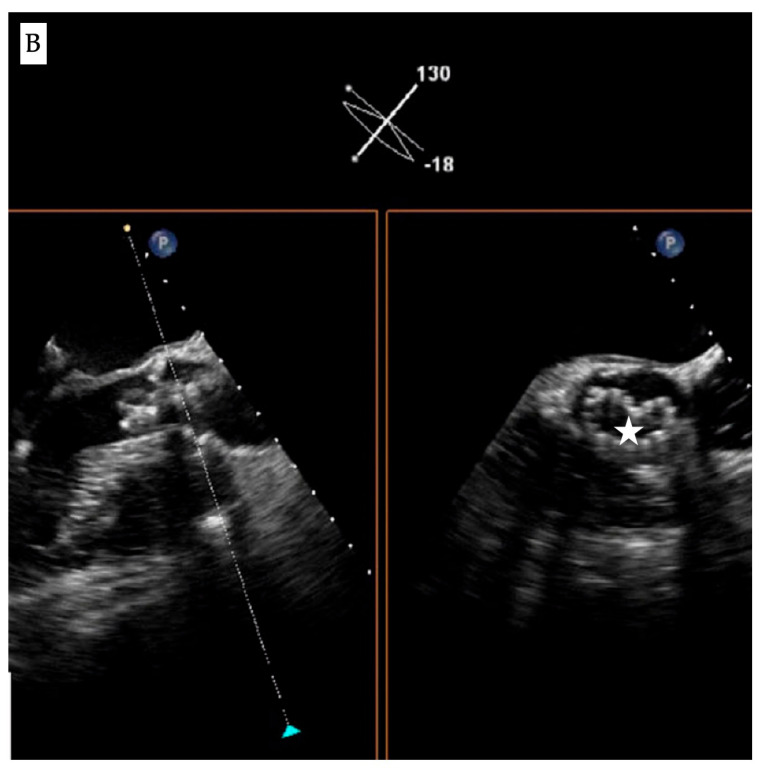
Fluoroscopic and echocardiographic appearance of TAVI infolding. (**A**) Visual clues for recognizing this complication include a dense vertical line across the valve frame (white arrow) and the inability to reach the intended valve expansion. (**B**) Short axis window in echocardiography revealing the “Pac man” sign (white star).

Once deployment has started, the two main fluoroscopic clues to identify it are (1) the appearance of a dense vertical line on the valve, reflecting the overlapping layers of the infolded stent cage, and (2) visual signs of *underexpansion* with a narrower-than-expected width [14] (Figure 1A).

Accordingly, evaluation of the bio-prosthesis in two orthogonal views or performing a c-arm fluoroscopic rotation is recommended to identify such complications. Infolding can also be confirmed by echocardiography through visualization of the “Pac-man” sign in the short axis view [15] (Figure 1B).

Several factors have been described as increasing the risk of TAVI infolding, as shown in Table 1.

Depending on the timing of the infolding diagnosis, there are different correction measures:

1. Before full release: The recommendation is to recapture, retrieve and reload the valve onto a new delivery catheter.
  If a high risk of infolding persists after several re-sheathing maneuvers, a more aggressive valve predilatation may be effective. Finally, switching to a BEV could be considered as a bail-out option.
2. After full release: Failure to identify this complication usually leads to hemodynamic instability due to severe aortic regurgitation. In this situation, urgent balloon post-dilatation is necessary to resolve the acute prosthetic dysfunction [18]. Careful manipulation of the guidewire is essential to avoid losing wire position and the risk of rewiring through the valve’s stent frame.
  A valve-in-valve implantation using a BEV or emergency cardiac surgery have been suggested as bail-out measures, if post-dilatation is insufficient to improve the patient’s clinical condition [11].

### 3.2. Guidewire Crossing Through an Inadequate Pathway

**Figure 2:**
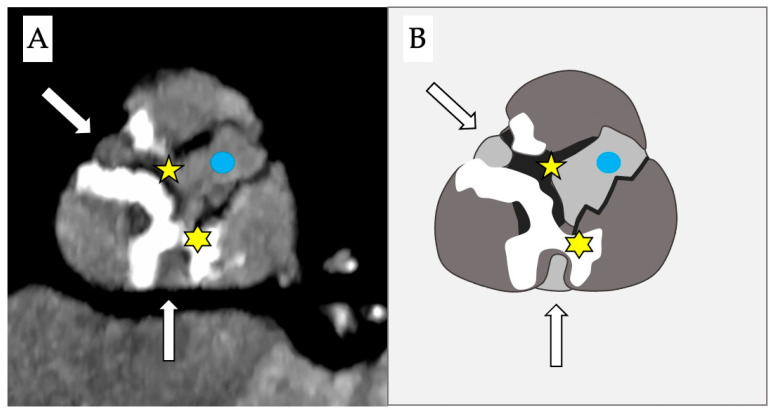
Computed tomography (**A**) and schematic figure (**B**) of a stenotic aortic valve that depicts the presence of 2 simultaneous bridges: one fibrotic between the right and the non-coronary cusps (yellow star) and a second calcific bridge between the left and the non-coronary cusps (yellow asterisk). This creates two anomalous new orifices (white arrows) where the guidewire can pass through and which are distinct from the true anatomical orifice (blue dot).

Stenotic disease of the aortic valve encompasses a wide range of anatomopathological variations, including the presence of fibrotic or calcific bridges between the cusps (Figure 2) that could allow passage of a guidewire through a lateral orifice.

Other possibilities are the presence of cusp perforations as a complication following endocarditis, or iatrogenic as sequalae from previous surgical interventions (e.g., aortic valvuloplasty) or due to percutaneous procedures (e.g., advancement of high-tip-load guidewires through the native cusp, complications from coronary atherectomy [19], etc.).

In this scenario, pre-dilating the native aortic valve without a clear understanding of the wire position can lead to severe aortic regurgitation and sudden onset of cardiac arrest [20].

**Figure 3:**
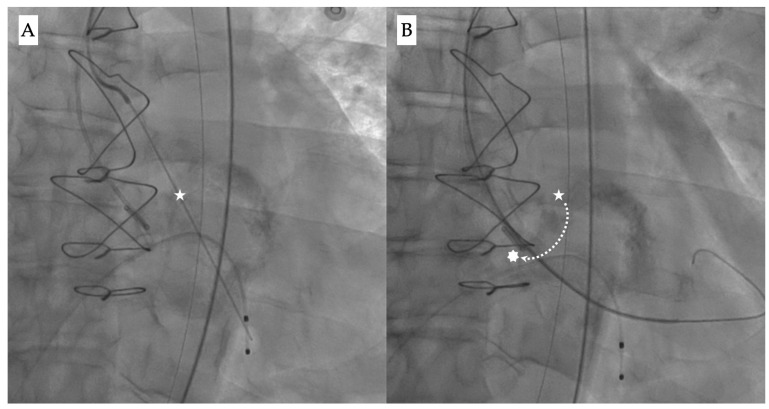
Fluoroscopy images showing (**A**) the initial position of the wire during valve crossing (white star), and (**B**) guidewire drop down (dotted line) from the center position into the inter-commissural space between the right and non-coronary cusp (white asterisk).

The “commissural drop” wiring technique may allow for a safer crossing during the procedure [21,22]. It describes the movement of the pre-shaped super stiff wire (e.g., Safari, Boston Scientific) from the center of the valve into the inter-commissural space between the non-coronary and right cusps, thus leaning towards the outer curvature of the aorta (Figure 3A,B). This is usually a sign of correct crossing through the central valvular orifice and provides a visual clue to confirm a safe position before proceeding.

If crossing the aortic valve is difficult, and in order to avoid losing wire position, another indirect technique could be attempting to advance an uninflated peripheral balloon through the guidewire in the left ventricle and checking for any resistance during advancement.

Finally, imaging techniques like transesophageal echocardiography could also help the operator assess the correct position of the guidewire.

### 3.3. True Underexpansion

This refers to an unsuccessful deployment due to mechanical factors that obstruct the expansion of the valve frame.

The most common one is severe calcification, which has been identified as one of the most important contributors, not only for underexpansion, but for complications such as annular rupture, need for pacemaker or PVL [23]. Contrary to non-contrast scans in which a fixed threshold of 130 Hounsfield units (HUs) is suggested, the threshold for calcium detection on contrast-enhanced CT scans has yet to be standardized in published reports. This lack of standardized acquisition parameters explains some of the difficulty in developing a uniform classification, although the range of 300–850 HU has been recommended [24]. CT is a fundamental part of the pre-planning phase and can help predict the risk of this complication, not only by estimating the amount of calcium, but also its distribution and characteristics (e.g., LVOT calcification, calcific nodules, etc.). This critical step can influence many decisions of the procedure such as THV selection, planning for upfront pre- and post-dilatation, depth of implant, need for coronary protection, pacemaker placement, etc.

Considering these issues, pre-dilatation is usually recommended in most bicuspid cases as it is for tri-leaflet valves with severe calcification measured by computed tomography (CT) scans (e.g., Agatston > 5000) or echocardiography. Operators must also be aware, especially in bicuspid anatomy, of the recoil phenomenon due to the fibro-calcific raphe, which enhances the difficulty of modifying the original “fish-mouth” shape.

During aortic valve-in-valve (ViV) procedures, there is also a risk of underexpansion.

One of the most critical points is to identify, whether with CT images or medical history, the type and size of the surgical prosthesis. Stentless surgical valves are similar to native aortic stenosis in terms of sizing at the virtual basal ring due to the absence of a rigid scaffold, but they are usually implanted in small root anatomies, which increases the risk for coronary occlusion [25]. In cases of stented prostheses, the direct measurement of the surgical internal diameter is a key component of proper sizing and avoiding mismatch. Also, the THV is anchored at the sewing ring but its expansion is also determined by the degree of calcification or pannus of the leaflets and the surgical posts. The depth of the implant is also relevant because a deeper deployment creates an overlap between the functional part of the surgical and transcatheter valves, thus increasing the risk of underexpansion [26].Taking into account the potential obstruction caused by the surgical posts, a low implantation would also be more likely to hinder proper expansion at the functional portion.

To mitigate this risk during ViV, careful pre-planning should be performed, including the implant depth and the possible need for post-dilatation or fracture of the surgical prosthesis [27]. This should also consider the potential risks involved as higher implantation can cause coronary obstruction or hinder coronary access and post-dilatation/fracture could lead to leaflet damage or increased risk of stroke [28].

**Figure 4:**
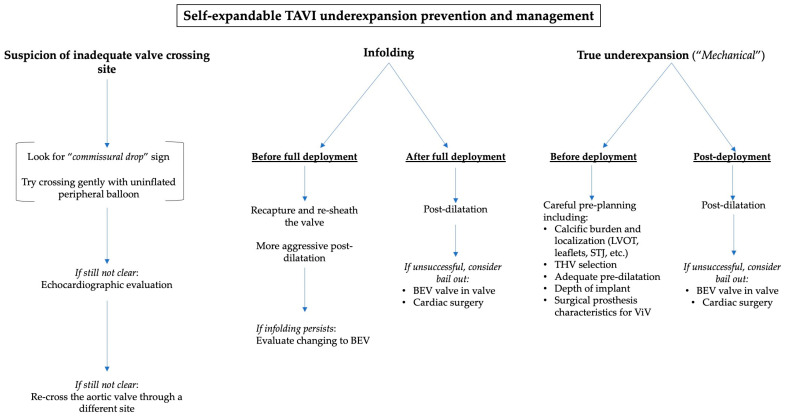
A practical algorithm to manage TAVI underexpansion depending on the suspected cause.

Figure 4 summarizes the main recommendations to avoid and to manage TAVI underexpansion.

## 4. Technical Considerations on Postdilatation

The decision to perform post-dilatation remains primarily based on functional assessments, such as the presence of paravalvular leak (PVL) and transvalvular gradients, which are frequently assessed by echocardiography. On the other hand, the use of angiographic features to guide post-dilatation remains less well-established, and further data are required to define clear criteria based solely on the fluoroscopic appearance of the prosthesis. Interestingly, some authors have proposed methods of evaluating underexpansion on BEVs [29] and SEVs [30], usually involving direct measurements of the valve frame and the use of different fluoroscopic views.

Proper balloon sizing is therefore critical as undersizing may fail to improve hemodynamics, while oversizing can lead to complications including leaflet damage, annular rupture, stroke or coronary artery occlusion [28]. To minimize these risks, safe post-dilatation should consider several key anatomical factors, including the presence of calcification at the left coronary cusp and the left ventricle outflow tract (LVOT). The bottom of the left cusp represents a point of less resistance and a higher risk of rupture compared to the right cusp, which is better “anatomically protected” by the presence of a muscular portion of the LVOT and is externally surrounded by the right ventricular outflow tract. Below the non-coronary cusp, there are the membranous septum and conduction bundles, particularly the atrioventricular node and the left bundle branch. Another important element is the size of the sinotubular junction, which is also a weak point and whose damage can lead to intramural hematoma or aortic dissection with the potential involvement of the coronary ostia [31]. In a recent study, LVOT calcium and STJ oversizing were the two strongest anatomical predictors of annular rupture when using BEV [32].

**Figure 5:**
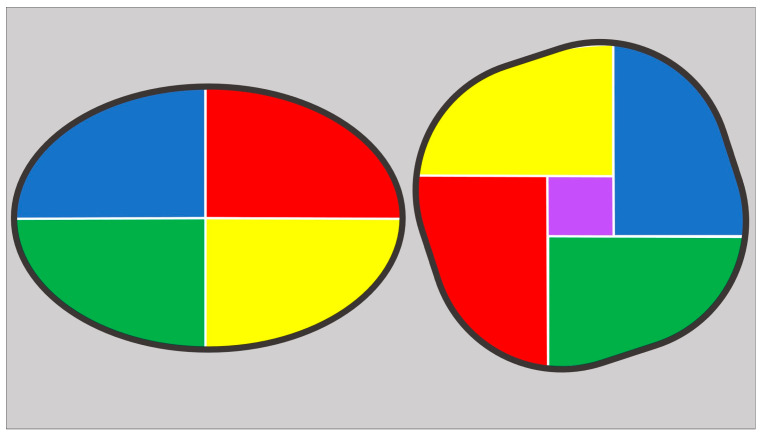
Ellipse (on the left) and circular figures (on the right) with the same perimeter. Despite having the same perimeter, the circular one has a larger area, as demonstrated by the presence of the purple square inside it. This increase in area when transitioning to a circle (while maintaining the same perimeter) is the effect achieved with the implantation of a BEV or with post-dilatation of an SEV.

Additionally, a “patient-tailored” approach is required to guide balloon sizing and ensure that the prosthesis is adequately expanded without exceeding the tolerance of the surrounding structures. This involves understanding the relationship between the perimeter and the area of the landing zone: two geometrical figures can have the same perimeter but different area, with the circle being the shape with the largest possible area (Figure 5). By choosing the balloon size based on the “area-derived diameter” instead of “perimeter-derived diameter”, just as is done with BEVs, we avoid the risk of oversizing when circularizing the virtual basal ring with the postdilatation [33].

## 5. Conclusions

In conclusion, TAVI underexpansion remains a significant concern, with various mechanisms contributing to its occurrence. Early detection and correction of these issues, coupled with careful procedural planning and execution, are essential for minimizing the associated risks. Future advancements in imaging and device technology may further reduce its incidence and help to improve long-term outcomes.

## Figures and Tables

**Table 1:** Risk factors for valve infolding *.

Anatomical Factors	Device-Related Factors
Eccentric or severe calcification of the native valve	Re-sheathing an SEV [16]
Type 1 bicuspid aortic valve	Inadequate valve loading
High ellipticity of the aortic annulus Severe tortuosity of the aorto-iliac and femoral artery	Larger valve sizes (e.g., ≥29 mm)

* Adapted from Karrowni [17].

## Data Availability

No new data were created or analyzed in this study.

## Abbreviations

The following abbreviations are used in this manuscript:

TAVI	Transcatheter aortic valve implantation
THV	Transcatheter heart valve
SEV	Self-expanding transcatheter valve
BEV	Balloon-expandable transcatheter valve
PVL	Paravalvular leak
HU	Hounsfield unit
ViV	Valve-in-valve
CT	Computed tomography
LVOT	Left ventricle outflow tract
CT	Computed tomography
